# An Overview of the Pathogenesis of Cutaneous Lupus Erythematosus

**DOI:** 10.3390/jcm14238285

**Published:** 2025-11-21

**Authors:** Alice Verdelli, Emanuela Barletta, Elena Biancamaria Mariotti, Simone Landini, Alessandro Magnatta, Valentina Ruffo di Calabria, Alberto Corrà, Lavinia Quintarelli, Irene Bonanni, Luca Sanna, Virginia Corti, Marzia Caproni

**Affiliations:** 1 Rare Skin Diseases Unit, P.O. Piero Palagi, Azienda USL Toscana Centro, European Reference Network-Skin Member, University of Florence, 50122 Florence, Italy; elenabiancamaria.mariotti@unifi.it (E.B.M.); irene.bonanni@gmail.com (I.B.); marzia.caproni@unifi.it (M.C.); 2 Department of Experimental and Clinical Biomedical Sciences “Mario Serio”, Experimental Pathology and Oncology Section, University of Florence, 50134 Firenze, Italy; emanuela.barletta@unifi.it; 3 Dermatology, Department of Health Sciences, University of Florence, 50125 Florence, Italy; simone.landini@unifi.it (S.L.); alessandro.magnatta@unifi.it (A.M.); valentina.ruffodicalabria@unifi.it (V.R.d.C.); lavinia.quintarelli@unifi.it (L.Q.); luca.sanna@unifi.it (L.S.); virginia.corti@unifi.it (V.C.); 4 U.O.C. Dermatologia, Ospedale San Bortolo, Azienda ULSS 8 Berica, 36100 Vicenza, Italy; alberto.corra@aulss8.veneto.it

**Keywords:** cutaneous lupus erythematosus, pathogenesis, keratinocytes, plasmacytoid dendritic cells, IFN, T cells, B cells, neutrophils, macrophages, drugs

## Abstract

**Background/Objectives:** Cutaneous lupus erythematosus (CLE) is a complex autoimmune skin disease driven by genetic predisposition, environmental triggers, and immune dysregulation. Environmental factors such as ultraviolet radiation, smoking, and certain drugs can initiate disease onset by inducing keratinocyte apoptosis. The subsequent release of nucleic acids and danger-associated molecular patterns activates pattern recognition receptors (PRRs) on keratinocytes and immune cells, leading to the production of type I and type III interferons (IFNs) and pro-inflammatory cytokines. The objective of this review is to summarize recent advances in understanding the immunopathogenesis of CLE, with particular attention to emerging cellular players and their therapeutic implications. **Methods:** A narrative review of the recent literature was performed, including experimental, translational, and clinical studies investigating the cellular and molecular mechanisms underlying CLE and novel targeted treatments derived from these findings. **Results:** Although plasmacytoid dendritic cells (pDCs) have traditionally been considered the major producers of IFN-I, recent data indicate that pDCs in CLE are functionally impaired and are not the primary source. Other cells, such as keratinocytes have emerged as key producers of IFN-I, contributing to a prelesional, IFN-rich microenvironment. This promotes the recruitment and activation of dendritic cells and other inflammatory myeloid subsets, which are now recognized as central players in amplifying local inflammation. Concurrently, T cells infiltrate the skin, where cytotoxic CD8^+^ T cells attack keratinocytes and CD4^+^ T cells further propagate inflammation via cytokine production. B cells and plasma cells produce autoantibodies, forming immune complexes that perpetuate inflammation. Neutrophils release neutrophil extracellular traps (NETs), exposing autoantigens and further stimulating IFN pathways. Macrophages contribute by presenting autoantigens, producing pro-inflammatory mediators, and failing to effectively clear apoptotic cells and immune complexes. **Conclusions:** The dynamic interplay between the innate and adaptive immune systems sustains the chronic inflammatory state characteristic of CLE. Based on the pathogenetic novelties, new therapeutic agents targeting specific molecules have been developed, which may improve the treatment of this complex disease in the future.

## 1. Introduction

**Figure 1:**
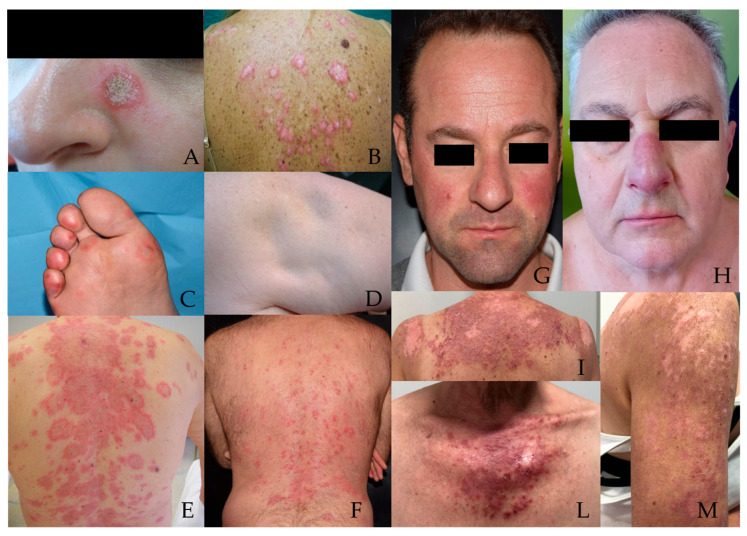
CLE subtypes (**A**) Localized DLE; (**B**) Generalized DLE; (**C**) ChLE; (**D**) LEP; (**E**) Annular SCLE; (**F**) Papulosquamous SCLE; (**G**) Localized ACLE; (**H**) LET; (**I**,**L**,**M**) Generalized ACLE. Abbreviation: ACLE: Acute Cutaneous Lupus Erythematosus; CLE: Cutaneous Lupus Erythematosus; ChLE: Chilblain Lupus Erythematosus; DLE: Discoid Lupus Erythematosus; LEP: Lupus Erythematosus Profundus; LET: Lupus Erythematosus Tumidus; SCLE: Subacute Cutaneous Lupus Erythematosus.

Cutaneous lupus erythematosus (CLE) is a heterogeneous autoimmune disease that may present as an exclusively cutaneous disorder or as part of systemic lupus erythematosus (SLE) [1]. Based on Gilliam and Sontheimer’s classification, CLE is divided into LE-specific and LE-nonspecific lesions [2]. LE-specific lesions include acute cutaneous LE (ACLE), subacute cutaneous LE (SCLE), and chronic cutaneous LE (CCLE). Specifically, ACLE encompasses localized forms, described as malar or “butterfly” rash, and, more rarely, a generalized form referred to as “maculopapular rash”. SCLE includes annular and papulosquamous (or psoriasiform) subtypes. CCLE encompasses localized and generalized discoid LE (DLE), LE profundus (LEP), and hypertrophic LE. It also includes chilblain LE (ChLE), a rare form characterized by painful or itchy red-purple lesions on cold-exposed areas like the fingers, toes, ears and nose. These lesions resemble chilblains (pernio) and often appear or worsen in cold, damp weather. LE tumidus (LET), initially considered a subtype of CCLE, has since been classified as a separate category, termed intermittent CLE (ICLE), although this distinction is not universally accepted [3] [Figure 1]. LE-nonspecific lesions, on the other hand, are not characteristic of LE but are frequently observed in active diseases. Such lesions include Raynaud’s phenomenon, periungual telangiectasias, livedo reticularis, and leukocytoclastic vasculitis [2].

The pathogenesis of CLE is complex, reflecting its clinical and immunological heterogeneity. It involves both innate and adaptive immune cells, triggered by environmental factors such as ultraviolet radiation (UVR), smoking, and drugs, in genetically predisposed patients [1]. Hormonal factors [4], metabolic dysregulation [5] and changes in the microbiome [6] may further modulate the inflammatory process.

### 1.1. Genetics

Most patients with CLE do not harbor specific genetic mutations, except for familial ChLE, a rare monogenic form caused by mutations in the *Three Prime Repair Exonuclease 1* (*TREX1*) gene, which encodes an enzyme responsible for cytosolic deoxyribonucleic acid (DNA) degradation [7].

However, numerous genetic variants have been associated with an increased risk of CLE, along with various epigenetic modifications [8].

Variants in *human leukocyte antigen* (*HLA*) *genes*, particularly in *HLA-DR3*, *HLA-B8*, and *HLA-DR2* region, as well as in *HLA-DQA1* alleles, are strongly linked to CLE [9,10,11].

Genetic overlap with SLE is evident, with shared susceptibility genes, although CLE-specific pathways remain distinct [12]. Polymorphisms in interferon (IFN) regulatory factor 5 (IRF5), cytotoxic T-lymphocyte-associated protein 4 (CTLA4), integrin alpha M (ITGAM), and tumor necrosis factor-alpha (TNF-α) have been reported [13,14,15]. Mutations or variants in genes such as *TREX1* [16], *IFN induced with helicase C domain 1* (*IFIH1*) [17], and tyrosine kinase (TYK2) [13] affect nucleic acid sensing and IFN pathways, contributing to chronic inflammation.

*TREX1* deficiency has also been associated with enhanced UV-induced DNA damage and heightened photosensitivity [18].

Epigenetic alterations, including aberrant DNA methylation and altered microRNA expression, have been implicated in CLE, further disrupting immune regulation [19,20].

### 1.2. UVR and Photosensitivity

UVR, particularly UVB, is a well-established environmental trigger in CLE, and plays a central role in both lesion initiation and propagation [21,22,23]. UVR exposure induces keratinocyte apoptosis and the translocation of nuclear autoantigens such as Ro/SSA and La/SSB. These autoantigens promote autoimmune responses and local skin inflammation [24,25]. UVR also enhances the production of IFN-I and pro-inflammatory cytokines, including interleukin (IL)-6, IL-1, and TNF-α, which together drive the recruitment and activation of pDCs and T lymphocytes in the skin [26,27].

In addition, UVR induces DNA photodamage, most notably through the formation of cyclobutane pyrimidine dimers (CPDs) [28]. CLE patients frequently display impaired DNA repair mechanisms and skin barrier dysfunction, making them more susceptible to UV-induced injury and prolonged immune activation. Barrier disruption facilitates deeper UV penetration and the entry of additional inflammatory stimuli, exacerbating cutaneous damage and sustaining chronic lesion development [29,30,31].

Recently, it has been demonstrated that patients with CLE and *TREX1* deficiency exhibit a UV-driven inflammation mediated by the cyclic GMP–AMP synthase (cGAS)–stimulator of IFN genes (STING) axis. Specifically, UV-induced DNA damage leads to the accumulation of reactive oxygen species (ROS), oxidative products (e.g., 8-oxo-guanine), and CPDs, which activate cGAS. This activation triggers downstream signaling through STING, TBK1, and IRF3, resulting in robust production of IFN-I [18], likely contributing to cutaneous flares in affected individuals. Current findings also implicate IFN-induced Z-DNA binding protein 1 (ZBP1) in this pathway: ZBP1 stabilizes UVB-induced cytosolic Z-DNA derived from oxidized mitochondrial DNA, amplifying IFN-I responses via cGAS–STING activation. ZBP1 is upregulated in the epidermis of photosensitive patients, highlighting its role as a key mediator of UVB-induced inflammation and a potential contributor to CLE photosensitivity [32].

Additionally, UVR exposure modulates the expression of matrix metalloproteinases (MMPs), which contribute to extracellular matrix remodeling and inflammatory amplification in CLE [33]. Precisely, MMP-1 and MMP-9 expressions are markedly upregulated in CLE skin following UVB irradiation [34]. This process appears to be mediated by keratinocyte-derived IL-15 and CCL5, which recruit mast cells that further enhance MMP secretion, establishing a pro-inflammatory amplification loop [35]. Elevated MMP-9 activity, measured by gelatin zymography, has been positively correlated with clinical severity scores (CLASI), especially in older patients and smokers [34].

Subtype-specific roles have also been described for MMP-28, particularly in ChLE. In these lesions, MMP-28 displays vertical dermal expression extending into the upper dermis, associated with decreased glucose transporte 1 (GLUT1) expression and dysregulated microRNAs, including miR-31 and miR-150 [33].

Despite the pathogenic importance of UVR, photoprovocation testing demonstrates variable sensitivity across CLE subtypes [21], ranging from 27 to 100% for SCLE, 25–90% for DLE, and 43–71% for LET [23]. Negative phototests do not rule out photosensitivity, as clinical history often diverges from test results [36]. This variability underscores the lack of standardized methods and precise definition of photosensitivity [37].

### 1.3. Metabolic Dysregulation in CLE

Emerging metabolomic data suggest that local metabolic imbalances in CLE skin contribute to lesion pathogenesis, particularly affecting energy-generating pathways [5]. A pioneering liquid chromatography-mass spectrometry (LC-MS) metabolomics analysis of lesional CLE skin revealed the significant depletion of nicotinamide adenine dinucleotide (NAD^+^)-related metabolites, implying impaired mitochondrial remodeling and adenosine triphosphate (ATP) production at the tissue level. These alterations were more pronounced in skin than in matched serum samples [5].

Although comprehensive skin-based metabolic studies are limited, insights from SLE models indicate that aberrant glycolytic activity, increased ROS production, and activation of mammalian Target of Rapamycin (mTOR) pathway may similarly affect immune and stromal cells within CLE lesions [38,39,40]. Recent lipidomic studies in SLE patients with skin involvement revealed alterations in sphingolipid species and vitamin E metabolites, which correlated with photosensitive phenotypes and anti-SSA seropositivity, suggesting a role for lipid metabolism in CLE subtype differentiation and disease severity [41,42]. Metabolomic profiling of patients with SLE and cutaneous involvement has also revealed consistent alterations in amino acid metabolism, including the cysteine/methionine, glutathione, and taurine/hypotaurine pathways, each implicated in redox homeostasis, inflammation, and immune regulation [43]. Notably, deficiencies in glutathione and methionine metabolism have been linked to elevated oxidative stress [44], a known amplifier of inflammatory responses in lupus [45]. These metabolic changes are not merely byproducts of inflammation but active drivers of immune cell function. pDCs exposed to altered redox environments produce higher levels of IFN-I [46,47], while metabolic stress in T cells can skew differentiation, reduce regulatory T cell function, and enhance effector responses [48,49]. These processes sustain a chronic inflammatory loop in the skin microenvironment.

Crucially, such metabolic abnormalities may persist in the absence of UV exposure, suggesting that they are intrinsic to disease maintenance rather than solely UV-triggered phenomena. This is supported by the data of lesional and non-lesional CLE skin, which show the upregulation of oxidative stress–related transcripts and IFN-stimulated genes (ISGs) regardless of recent sun exposure [50]. Combined transcriptomic and machine learning approaches have demonstrated that non-lesional CLE skin exhibits distinct immune activation signatures alongside suppression of oxidative phosphorylation and lipid metabolism pathways, highlighting subclinical metabolic reprogramming [51].

Moreover, these abnormalities are not confined to UV-exposed sites, as non-lesional, sun-protected anatomical areas still exhibit an IFN-rich and metabolically dysregulated environment [51]. Additionally, immune–stromal crosstalk has been implicated in sustaining local inflammatory circuits in CLE skin, potentially contributing to photosensitivity and chronic lesion persistence, even in the absence of direct UV activation [52].

These findings position metabolic dysregulation as an additional, non-environmental axis of CLE pathogenesis, and highlight it as a potential therapeutic target.

### 1.4. Smoking

Smoking plays a multifaceted role in CLE, being associated with increased disease activity [53,54,55] and reduced treatment efficacy [56,57,58]. Multiple studies have reported a higher prevalence of smoking among patients with CLE, particularly those with DLE [59,60], SCLE [61], and LET [54,62]. Smokers often present with more severe and persistent skin lesions and report a poorer quality of life compared with non-smokers [54,63].

Smoking is also linked to a diminished response to hydroxychloroquine [56,57,58], the first-line treatment for CLE [64]. The mechanisms underlying this reduced efficacy remain unclear. Possible explanations include direct interactions between cigarette smoke and medication, higher baseline disease severity in smokers, or reduced treatment adherence. Indeed, smokers are often less likely to follow medical recommendations consistently [65,66]. Collectively, these findings identify smoking as a key modifiable risk factor in CLE management.

### 1.5. Drugs

Many drugs can induce CLE, including thiazide diuretics, calcium channel blockers, proton pump inhibitors, terbinafine, anti-TNF-α agents, antiepileptics, and certain chemotherapeutics like 5-fluorouracil or taxanes [67,68,69,70]. Specific genetic factors, such as certain *HLA* alleles and complement components, may predispose individuals to developing drug-induced LE (DILE) by enhancing their immune response to medications [71,72]. The precise mechanisms underlying pathogenesis remain incompletely understood but are thought to involve several distinct immunological processes, with four principal hypotheses proposed to account for disease development [73]. One proposed mechanism is hapten formation, whereby a drug or its metabolite binds to self-proteins and generates a novel antigen that may elicit an autoimmune response through molecular mimicry [74]. Another plausible process involves direct cytotoxicity by reactive metabolites, which damage cells and provoke secondary immune activation [75,76,77]. Certain drugs may also disrupt central immune tolerance by impairing thymic selection and permitting autoreactive T cells to escape deletion [78,79]. In addition, many implicated drugs can alter immune tolerance through epigenetic modifications, particularly by inhibiting DNA methylation in CD4^+^ T cells [80,81].

Recent findings suggest that neutrophil extracellular traps (NET)osis, a specialized form of neutrophil cell death involving the release of DNA-rich extracellular traps, plays a role in DILE. Lupus-inducing drugs like procainamide and hydralazine have been shown to promote NET formation through distinct pathways [82].

Lupus-like symptoms have been reported following initiation of TNF inhibitors (e.g., infliximab, etanercept), although a causal relationship remains difficult to establish. It is unclear whether TNF inhibitors induce de novo lupus or simply unmask preexisting, subclinical lupus in genetically or immunologically predisposed individuals [83,84,85,86]. Many patients receiving TNF blockers develop antinuclear antibodies (ANA) or anti-dsDNA antibodies; however, most do not progress to clinical lupus [87,88].

Several mechanisms have been proposed to explain TNF inhibitor–induced lupus, though none have been definitively established. These include: (1) an imbalance between TNF-α and IFN-α due to enhanced pDCs activity [85]; (2) accumulation of apoptotic debris, such as nucleosomes, triggering autoantibody production [84]; (3) a shift from Th1 to Th2 immune responses [89]; and (4) increased susceptibility to infections, promoting B-cell activation and transient autoantibody formation [90].

TNF inhibitor-induced lupus often resolves after drug discontinuation, supporting a drug-related effect [91]. However, this does not exclude the possibility that the TNF blockade merely revealed an underlying predisposition to lupus. Current evidence favors the hypothesis that TNF inhibitors may unmask latent lupus rather than directly causing the disease, although further studies are needed to clarify their role [91,92].

More recently, lupus has also been reported in association with immune checkpoint inhibitors (ICIs) [93,94]. It remains uncertain whether this represents a novel immune-mediated cutaneous toxicity or the reactivation of a previously silent autoimmune condition. A proposed “multi-hit” model suggests that ICIs may act as an additional trigger, amplifying immune responses to antigens that were previously well tolerated [95].

### 1.6. Hormones

Sex hormones play a key role in the pathogenesis of SLE, but their impact on CLE, particularly DLE, appears less pronounced [4,96]. Nevertheless, CLE remains more common in women, even outside the fertile period, suggesting that additional factors contribute to the higher incidence in females [97]. The absence of significant disease exacerbation during pregnancy or with oral contraceptive use further supports this notion.

### 1.7. Microbiome

The role of the cutaneous microbiota in CLE pathogenesis remains unclear. Alterations in microbial populations, such as increased *Staphylococcus* and *Corynebacterium* and decreased *Cutibacterium*, have been observed in SLE skin lesions, although no direct evidence has yet linked these changes to CLE [30,98]. Further research in this area is warranted.

### 1.8. Cancers

CLE has occasionally been linked to malignancy, particularly lymphoma, nonmelanoma skin cancer, buccal cancer, and lung cancer. Among CLE subtypes, SCLE appears to be the most frequently associated with malignancy [99].

## 2. Cellular and Immunopathological Features Across CLE Subtypes

Overall, both exogenous and endogenous triggers contribute to keratinocyte death and the subsequent release of cellular debris, which activate danger-associated receptors and recruit inflammatory cells [24,100,101]. A key feature in the development of CLE is the overexpression of IFNs, particularly IFN-α, which establishes an inflammatory loop resembling an antiviral response. Elevated levels of IFN-α contribute to inflammation, immune dysregulation, and lesion formation in CLE [102,103,104]. Although autoreactive T cells and pDCs have traditionally been considered central to disease pathogenesis [103,105,106,107], recent evidence indicates that keratinocytes, B cells, neutrophils, and macrophages also play a significant role [108,109,110] [Table 1].

All CLE subtypes share common histopathological features; however, their clinical manifestations and underlying immune pathways differ, as reflected by variations in autoantibody profiles and immune cell composition [111]. Recent immunohistochemical analysis have demonstrated distinct lesional cellular infiltrates in CLE that vary according to subtype and disease stage [112,113,114,115]. The inflammatory infiltrate may be dominated by pDCs, B cells, T cells, or characterized by a strong IFN-I signature.

DLE lesions typically exhibit prominent cytotoxic CD8^+^ T-cell infiltration in the early phase, while CD20^+^ B cells predominate in later stages [116,117]. These infiltrates are accompanied by M1 macrophages and fibrotic remodeling processes involving TGF-β and MMP-9, which contribute to scarring [118]. SCLE lesions, by contrast, display a mixed CD4^+^/CD8^+^ T-cell infiltrate, elevated IL-17 expression, and moderate inflammatory infiltrates, with less fibrosis and fewer B cell infiltrates compared to DLE [117,119].

ACLE, often associated with SLE [2], is characterized by highly inflammatory lesions dominated by CD4^+^ T cells and type I IFN-driven pathways. Notably, no immunohistochemical studies have focused exclusively on ACLE; current knowledge is based on analyses of mixed CLE subtypes and transcriptomic studies that include ACLE samples [112,120].

LET exhibits the highest level of pDC infiltration among CLE subtypes [121].

Further studies are warranted to elucidate the distinct immunopathological phenotypes of CLE subtypes.

The following sections will discuss the roles of individual immune cell populations in greater detail.

## 3. The Role of Keratinocytes in CLE

Keratinocytes, the predominant epidermal cell type, are central orchestrators of CLE pathogenesis. As previously described, upon exposure to environmental or endogenous stressors—including but not limited to UVR, smoking, or certain drugs—they release nuclear and cytoplasmic autoantigens, generate ROS, and secrete cytokines and chemokines that recruit and activate immune cells [24,25,101,111].

Defective clearance of apoptotic debris and nucleic acids allows the accumulation of self–RNA and DNA [24,101], which are sensed by keratinocytes and antigen-presenting cells (APCs) via pattern recognition receptors (PRRs), including melanoma differentiation-associated protein 5 (MDA5), retinoic acid-inducible gene (RIG-I), and cGAS–STING [101]. This recognition drives IFN-regulated gene expression through toll-like receptors (TLR)-independent mechanisms [122].

Keratinocytes secrete IFNĸ and IFNλ (types I and III IFNs), which induce the production of pro-inflammatory cytokines (e.g., IL-6, TNF-α) and chemokines (e.g., CXCL9, CXCL10, and CXCL11), thereby amplifying immune activation [123,124]. At the same time, IFN-I enhances PRR expression in keratinocytes, creating a cycle of chronic inflammation [125]. Chemokines secreted by keratinocytes attract T cells, macrophages, and pDCs to the skin, perpetuating inflammation. Keratinocytes also present antigens to T cells via MHC molecules, directly promoting immune activation [107]. Activated CD8^+^ T cells kill keratinocytes, contributing to epidermal damage observed in CLE lesions [103].

Keratinocyte death in CLE occurs through multiple, overlapping pathways whose relative contributions are shaped by the cytokine milieu and tissue context. Type I IFN priming, particularly via keratinocyte-derived IFNκ, selectively enhances caspase-8–dependent apoptosis after UVB exposure, as demonstrated in IFNk-overexpressing murine skin and in vitro keratinocyte models [126]. This effect is mediated by IFN-induced upregulation of IRF1 and is independent of classical death ligands.

In contrast, IFN-γ and TNF-α, often derived from activated T cells in lesional skin, can drive keratinocyte necroptosis—a lytic form of programmed cell death—via the phosphorylation of receptor-interacting-protein-kinase 3 (RIPK3) and mixed lineage kinase domain like pseudokinase (MLKL) [127]. Necroptosis leads to the release of damage-associated molecular patterns (DAMPs) [128] such as HMGB1, thereby amplifying local inflammation and potentially perpetuating autoimmunity [129,130]. Both apoptosis and necroptosis are likely to contribute to the interface dermatitis pattern seen in CLE.

Beyond established lesions, recent single-cell transcriptomic analysis revealed that clinically normal-appearing, non-lesional CLE skin exists in a type I IFN–primed “prelesional” state [50]. At the interfollicular dermo-epidermal junction, keratinocytes serve as a major source of IFN, reprogramming surrounding stromal and immune cells and promoting the recruitment of CD16^+^ dendritic cells—potentially seeding sites for future lesion development. This ability to sustain IFN signaling and present antigens via MHC molecules positions keratinocytes as active participants in immune activation, rather than passive targets of injury.

## 4. The Role of pDCs in CLE

pDCs have long been considered central to lupus pathogenesis due to their capacity to produce large amounts of IFN-I, particularly IFN-α, in response to nucleic acids sensed via TLR7 and TLR9 [131,132,133].

In CLE, pDCs are recruited to skin lesions through chemokine interactions—such as CXCL10 binding to CXCR3—and often form cellular clusters within lesional sites [100,134]. Their robust IFN-I production was historically thought to initiate and sustain a self-amplifying inflammatory loop [135]. However, not all lesions harbor pDC-rich infiltrates, and their density may vary across different CLE subtypes [112,136]. An IFN-rich environment primed by pDCs and other dendritic cell subsets has also been detected in the non-lesional skin of CLE patients [50].

Recent findings suggest that pDCs are not the primary source of IFN-I in lupus. In fact, pDCs from CLE patients produce significantly less IFN-α than those from healthy donors, indicating functional exhaustion [137,138]. Instead, keratinocytes, monocytes/macrophages, and conventional dendritic cells have emerged as dominant contributors to local IFN-I activity [123,138,139].

Despite diminished IFN production, pDCs may still exert pathogenic effects through non-canonical mechanisms. Notably, they express granzyme B (GZMB), a serine protease implicated in keratinocyte apoptosis and immune modulation [140]. GZMB^+^ pDCs colocalize with cytotoxic T and natural killer (NK) cells at sites of keratinocyte death, and their presence correlates with inflammatory damage in CLE lesions. Whether GZMB production directly drives pathogenesis or represents a compensatory regulatory mechanism remains unclear.

Insights into pDCs biology have also informed therapeutic strategies. Blood dendritic cell antigen 2 (BDCA2), a receptor expressed on pDCs, is a target of experimental treatments. Litifilimab, a humanized monoclonal antibody targeting BDCA-2, demonstrated clinical efficacy in phase 2 trials for CLE and reduced expression of Myxovirus resistance protein A (MxA) in responsive patients, suggesting downstream modulation of IFN-I [141,142]. However, recent data indicate that BDCA2 is also expressed on pro-inflammatory monocytes [143], raising questions about the precise mechanism underlying therapeutic benefit.

Overall, the evolving understanding of pDCs in CLE emphasizes their heterogeneous roles, shifting from principal IFN producers to context-dependent contributors to local immune pathology. These insights underscore the need for precision therapies that account for cellular and molecular diversity within CLE lesions.

## 5. IFN: The Role of the JAK/STAT Pathway

The Janus kinase (JAK)/signal transducer and the activator of the transcription (STAT) pathway is a key mediator of the inflammatory response triggered by IFNs, particularly IFN-α and IFN-β. Upon binding to their receptor (IFNAR) on keratinocytes and immune cells, JAK1 and TYK2 are activated and phosphorylate specific signal transducers and activators of transcription (STAT1 and STAT2). These phosphorylated STATs form a complex with IRF9, known as ISGF3, which translocate to the nucleus and induces IFN-stimulated genes (ISGs) [144]. ISG expression promotes immune cell recruitment and activation, amplifies the IFN response, and upregulates inflammatory mediators, thereby perpetuating skin inflammation and tissue damage. Sustained JAK-STAT activation by IFNs is a hallmark of CLE and represents a promising therapeutic target [145].

Several clinical investigations have examined JAK inhibition in CLE, yielding mixed but encouraging results. Baricitinib, a JAK1/2 inhibitor, improved skin symptoms in the Phase III SLE-BRAVE I trial; however, these findings were not replicated in SLE-BRAVE II [146]. In a small, randomized cohort of five patients with LEP, baricitinib led to significant improvement in disease activity in two of the three treated patients [147].

Tofacitinib, a JAK1/3 inhibitor, achieved partial efficacy in a small Phase II pilot study in DLE, although recruitment challenges limited its evaluation [148]. Filgotinib, a selective JAK1 inhibitor, failed to meet its primary endpoint in a Phase II trial for moderate-to-severe CLE [149]. In contrast, deucravacitinib, a selective TYK2 inhibitor, has shown superior efficacy, significantly improving CLE symptoms [150].

Mechanistic research demonstrated that TYK2 is highly expressed in CLE lesions and closely associated with interface dermatitis. In vitro and ex vivo experiments using keratinocytes, three-dimensional skin models, CLE T cells, and skin biopsies revealed that TYK2 inhibition suppresses IFN-driven inflammation, restores epidermal integrity, and downregulates necroptosis-related gene expression.

Importantly, in four patients with therapy-refractory CLE across multiple subtypes, deucravacitinib achieved marked clinical improvement, underscoring its therapeutic potential [151]. Additionally, topical ruxolitinib (JAK1/2 inhibitor) has shown benefit in individual cases, suggesting a possible role for local JAK inhibition in CLE management [152].

## 6. The Role of T Cells in CLE

T cells, particularly CD4^+^ and CD8^+^ subsets, are central to the CLE pathogenesis, driving inflammation, keratinocyte apoptosis, and chronic tissue damage [153].

Lesional infiltrates are dominated by T cells, together with B cells, dendritic cells, NK cells, and occasional neutrophils [103,154]. Recruitment of CXCR3-expressing T cells to the skin is driven by chemokines such as CXCL10, produced by keratinocytes and other immune cells, establishing a pro-inflammatory microenvironment [103].

Following antigen presentation by APCs, T-cell receptor (TCR) engagement triggers signaling cascades that promote effector functions [155].

Cytotoxic CD8^+^ T cells target basal keratinocytes, producing the interface dermatitis characteristic of CLE. Granzyme B, induced by type I IFNs, is particularly enriched in scarring discoid lesions compared with non-scarring subacute lesions, implicating it in irreversible tissue injury [156]. CD4^+^ T cells contribute to keratinocyte apoptosis via Fas/Fas ligand (FasL) interactions with IFN-γ–stimulated keratinocytes and secrete IL-21, which induces granzyme B production in pDCs and enhances NK-cell cytotoxicity [140,157].

Early CLE lesions may display Th2-polarized inflammation, whereas chronic lesions are dominated by Th1 cells. High IFN-γ levels further recruit cytotoxic lymphocytes and activate macrophages, amplifying tissue damage [158].

The balance between effector and regulatory T cells (Tregs) is also altered in CLE. Tregs, including FOXP3^+^ subsets, are numerically and functionally reduced, especially in photosensitive forms such as SCLE and LET, while SCLE lesions show lower CD4/CD8 ratios and fewer Tregs compared with DLE [12,159,160].

Dermal CD4^+^ tissue-resident memory T (Trm) cells also contribute to disease persistence. These cells are more abundant in SCLE and localized DLE lesions than in ACLE, and expression of absent in melanoma 2 (AIM2) within CD4^+^ Trm cells is markedly higher in SCLE and DLE. AIM2 quantification can distinguish ACLE from these chronic subtypes with high sensitivity and specificity, highlighting its potential as a biomarker of CLE phenotype [161].

Pathogenic T-cell activity is amplified by aberrant intracellular signaling. CLE-infiltrating CD4^+^ and CD8^+^ T cells upregulate hypoxia-inducible factor 1 (HIF-1), enabling adaptation to hypoxia and sustaining a strong cytotoxic transcriptional program. Inhibition of HIF-1 in murine lupus skin disease reduces both cutaneous and systemic pathology by suppressing T-cell cytotoxicity [162]. In addition, spleen tyrosine kinase (SYK) and its phosphorylated form (pSYK) are markedly elevated in CLE lesions, particularly in keratinocytes and infiltrating immune cells, and are induced by immunostimulatory nucleic acids [163]. SYK activation promotes pro-inflammatory cytokine production via NF-κB and related pathways, while small-molecule SYK inhibitors suppress these responses in vitro [164]. Despite this mechanistic rationale, clinical translation remains challenging: in a Phase Ib trial, the topical SYK inhibitor GSK2646264 was well tolerated but produced only modest reductions in IFN-related gene expression and immune cell markers, without clear clinical benefit [165].

Together, these findings highlight the multifaceted role of T cells in CLE—as direct mediators of keratinocyte injury, modulators of chronic inflammation, and amplifiers of pathogenic signaling—and underscore the complexity of translating mechanistic insights into effective therapies.

## 7. The Role of B Cells in CLE

B cells play a multifaceted role in the pathogenesis of CLE, contributing to disease progression through autoantibody production, antigen presentation, cytokine release, and interactions with T cells. Autoantibodies, such as anti-Ro and anti-La antibodies, frequently detected in CLE and SLE [112,166], bind nuclear material released from apoptotic keratinocytes to form immune complexes that sustain inflammation and tissue damage. Prolonged exposure to IFN-I amplifies this response by enhancing B-cell survival, activation, and autoantibody production [167]. Beyond their classical antibody-mediated functions, B cells act as APCs, activating autoreactive T-cell subsets and perpetuating cutaneous inflammation [1,97].

A recent murine CLE model highlighted the importance of IL-21 and TLR7/TLR9 signaling in promoting B-cell recruitment to skin lesions and in supporting local autoantibody production, suggesting that lesional B-cell responses may occur independently of systemic autoimmunity [168].

B cells additionally secrete pro-inflammatory cytokines such as IL-6, which enhances B-cell survival, promotes autoantibody production [112,169], and contributes to the chronic inflammatory environment in CLE.

Importantly, strong B-cell transcriptional signatures and lesional B-cell infiltrates have been demonstrated even in autoantibody-negative CLE patients [109,112], shifting the focus from systemic to cutaneous B-cell immunity.

Lesional CLE skin often contains B cells infiltrates that cluster to form tertiary lymphoid structures, particularly in subtypes such as DLE and LEP. These structures promote local immune responses and perpetuate autoimmunity [112,117].

Keratinocytes in CLE lesions express B-cell activating factor (BAFF), a cytokine known to support B-cell maturation and survival in other contexts. Although a direct functional interaction between keratinocyte-derived BAFF and B cells within CLE have not yet been demonstrated, its expression suggests a potential role in shaping the local immune milieu [170].

While B cells are primarily recognized for their pro-inflammatory activity in CLE, regulatory B-cell functions have been described in SLE and other autoimmune settings [171]. Whether a similar dual immunomodulatory role exists in skin remains to be investigated in CLE.

Given their pathogenic importance, B cells have emerged as therapeutic targets in CLE. BAFF inhibitors such as belimumab have shown efficacy in reducing CLE disease activity in clinical studies, though they are not specifically approved for CLE [172]. Belimumab improves overall disease activity in SLE, including cutaneous manifestations, and may be particularly beneficial in DLE [173]. CD20-based B-cell depletion therapies (e.g., rituximab) are also being explored for refractory cases, but their efficacy appears variable across CLE subtypes [174,175].

## 8. The Role of Neutrophils in CLE

Neutrophils contribute to both the acute and chronic inflammation observed in CLE. Their ability to form NETs, release pro-inflammatory cytokines and enzymes, and interact with other immune cells positions them as central mediators of cutaneous inflammation [110,176].

NETs are markedly increased in CLE skin lesions, including ACLE, DLE, and LEP. By exposing DNA, chromatin, LL-37 and other nuclear/intercellular components, NETs act as a source of autoantigens [177].

Neutrophils are frequently detected infiltrating the skin of patients with CLE, particularly in the acute forms. In response to inflammatory signals, they migrate from the circulation info affected tissues, where they contribute to lesion formation. These infiltrates are often localized in perivascular regions, a hallmark of the inflammatory response in CLE [110]. Recruitment of neutrophils to the skin is mediated by pro-inflammatory cytokines and chemokines, such as TNF-α, IL-8, and CXCL8, which are elevated in lupus lesions [178]. Environmental triggers such as UVR further enhance neutrophil activity. UV promotes the release of inflammatory cytokines that activate and recruit neutrophils, contributing to disease flares [179]. One recruited, neutrophils interact with T cells, B cells, and DCs, amplifying the autoimmune response [180].

In addition to releasing cytokines, neutrophils influence the activation of autoantibody-producing B cells and participate in immune complex formation and deposition, processes that sustain inflammation and tissue injury [180].

Sustained neutrophil activation damages the dermal–epidermal junction and contributes to disease chronicity. Neutrophil-mediated vascular damage and hypoxia further exacerbate tissue injury in CLE [181]. Understanding the mechanisms by which neutrophils promote tissue damage and immune dysregulation may enable the development of therapeutic strategies targeting these cells.

## 9. The Role of Macrophages

Macrophages contribute to the pathogenesis and progression of SLE by exhibiting a reduced ability to transition from the pro-inflammatory M1 state to the anti-inflammatory M2 phenotype, a shift that is critical for tissue repair [182]. While research specifically addressing macrophage function in CLE is limited, emerging evidence suggests that they also play important roles in this disease.

In SLE, monocytes demonstrate enhanced antigen-presenting activity [183], impaired clearance of apoptotic material, and prolonged phagocytic engagement, collectively leading to the accumulation of autoantigens and sustained immune activation [184,185]. Macrophages from lupus patients also show defective efferocytosis and adhesion, particularly in the presence of lupus serum [185], favoring local immune complex deposition and inflammation.

Importantly, a mechanistic study of UVB-induced lesions revealed that, although the rate of apoptotic keratinocyte clearance is comparable between SLE patients and healthy controls, lupus skin shows a qualitatively distinct macrophage response. Following UV irradiation, lupus patients display a significantly greater influx of macrophages into both the dermis and epidermis, especially at sites where inflammatory lesions develop [186]. These infiltrates cluster around apoptotic keratinocytes and are associated with increased T-cells recruitment, suggesting that macrophages engage in a pro-inflammatory mode of apoptotic cell clearance rather than a failure of phagocytosis per se. This “inflammatory efferocytosis” mirrors the pathology of spontaneous CLE lesions and implies that macrophages may drive cutaneous inflammation by responding abnormally to physiological levels of apoptotic cells, thereby amplifying local immune activation instead of resolving it.

Histological and transcriptomic analyses further support the involvement of macrophages in CLE. Single-cell RNA sequencing skin lesions from patients with DLE and SCLE demonstrated increased proportions of macrophage/dendritic cell populations in the dermis compared with healthy controls [82]. Notably, FasL expressing macrophages have been identified around hair follicles in DLE and LEP, where they may contribute to scarring alopecia via Fas/FasL-mediated keratinocyte apoptosis [187].

A clinical trial assessing PD-0360324, a monoclonal antibody against macrophage colony-stimulating factor (M-CSF), demonstrated suppression of circulating monocytes and altered activity of some tissue macrophages in CLE patients. However, the treatment did not significantly affect macrophage populations in lesional skin or improve clinical outcomes, highlighting the complexity of macrophage involvement in CLE [188].

## 10. Conclusions

CLE is a complex condition shaped by interactions among multiple immune cell populations and cytokine networks. Distinct immune cell infiltrates, cytokine expression patterns, and tissue remodeling processes underlie differences between CLE subtypes [97]. Recent immunohistochemical analyses have revealed subtype-specific immune signatures based on disease severity and dysregulated pathways [108,109,110,111,112]. These differences influence disease course, therapeutic response, and potential treatment targets. Advancing personalized medicine in CLE will require a deeper understanding of the molecular mechanisms and individual triggers driving disease heterogeneity.

## 11. Future Directions

A deeper understanding of immune dysregulation in lupus is driving the development of therapies targeting specific immune pathways. Promising strategies include B-cell–directed therapies, T-cell modulators, cytokine inhibitors, and small-molecule inhibitors of intracellular signaling pathways. Ongoing and future clinical trials will be crucial to translating mechanistic insights into effective, personalized treatments for CLE.

## Figures and Tables

**Table 1:** Key genetic factors, environmental triggers, and immunopathogenic mechanisms in CLE.

Genetic Factors	Triggers	Cells/Pathways
***HLA* polymorphisms** (*HLA-DR3, HLA-B8, HLA-DR2, HLA-DQA1*) **TREX1, IRF5, ITGAM, TYK2 mutations** **TREX1 deficiency** **Epigenetic alterations** (DNA hypomethylation, microRNAs)	**Ultraviolet (UV) radiation:** ○ Keratinocyte apoptosis ○ Autoantigen release ○ cGAS–STING and ZBP1 activation ○ Enhance type I IFN production ○ MMPs upregulation **Metabolic dysregulation:** ○ NAD^+^ depletion ○ ROS increase ○ mTOR activation ○ Altered amino acid/lipid metabolism **Smoking:** ○ Increased disease severity ○ Reduced antimalarial efficacy **Drugs:** ○ Molecular mimicry ○ Cytotoxicity ○ Impaired tolerance ○ Epigenetic effects **Microbiota:** ○ Altered skin composition	**Keratinocytes:** ○ Apoptosis/necroptosis ○ IFNκ/IFNλ production ○ Cytokines production (e.g., IL-6 and TNF-α) ○ Chemochines production (e.g., CXCL9-11) ○ Antigen presentation **Plasmacytoid dendritic cells:** ○ TLR7/9 sensing ○ Type I/III IFN production ○ Granzyme B (GZMB) expression ○ Impaired in CLE **JAK/STAT pathway:** ○ IFN-driven inflammation ○ JAK1/TYK2 activation ○ STAT1/STAT2→ISGs **CD4^+^ and CD8^+^ T lymphocytes:** ○ Main lesional cells ○ Cytotoxic CD8^+^ (interface dermatitis) ○ Fas/FasL-mediated keratinocytes apoptosis ○ Early Phase: Th2-polarized inflammation ○ Later-phase: Th1 cells ○ Impaired Tregs ○ Elevated SYK expression ○ Dermal CD4^+^+Trm cells: disease persistence **B lymphocytes:** ○ Autoantibody production ○ Immune complexes ○ APC role ○ Tertiary lymphoid structures formation ○ BAFF interaction **Neutrophils:** ○ NETosis ○ Increased NETs **Macrophages:** ○ FasL^+^ macrophages

## Data Availability

The original contributions presented in this study are included in the article. Further inquiries can be directed to the corresponding author.
